# The effects of gender-affirming hormone therapy and mastectomy on psychopathology, body image, and quality of life in adults with gender dysphoria who were assigned female at birth

**DOI:** 10.1007/s11136-024-03664-6

**Published:** 2024-04-24

**Authors:** Şenol Turan, Mahmut Taha Özulucan, Uğur Karataş, Yasin Kavla, Oğuzhan Koyuncu, Emre Durcan, Gizem Durcan, Semih Bağhaki

**Affiliations:** 1 Department of Psychiatry, Cerrahpasa Medical Faculty, Istanbul University-Cerrahpasa, Istanbul, Türkiye; 2 Graduate School of Health Science, Neuroscience PhD Program, Koç University, Istanbul, Türkiye; 3 Cerrahpasa Medical Faculty, Istanbul University-Cerrahpasa, Istanbul, Türkiye; 4 Department of Psychiatry, Hınıs State Hospital, Erzurum, Türkiye; 5 Department of Child and Adolescent Psychiatry, Medeniyet University, Istanbul, Türkiye; 6 Division of Endocrinology and Metabolism, Department of Internal Medicine, Cerrahpasa Medical Faculty, Istanbul University-Cerrahpasa, Istanbul, Türkiye; 7 Department of Child and Adolescent Psychiatry, Istanbul University-Cerrahpasa, Istanbul, Türkiye; 8 Department of Plastic, Reconstructive and Aesthetic Surgery, Cerrahpasa Medical Faculty, Istanbul University-Cerrahpasa, Istanbul, Türkiye

**Keywords:** Gender dysphoria, Psychopathology, Body uneasiness, Quality of life, Gender-affirming hormone therapy, Mastectomy

## Abstract

**Purpose:**

Individuals with gender dysphoria (GD) may request hormone therapy and various surgical operations to change their physical characteristics. The present study aimed to investigate the effects of two treatments, mastectomy and gender-affirming hormone therapy (GAHT), on adults with GD who were assigned female at birth (GD AFAB).

**Methods:**

In this cross-sectional study, we gathered data from a total of 269 individuals in three groups: (a) untreated group (*n* = 121), (b) GAHT group (*n* = 84) who had been receiving treatment for at least 6 months, and (c) GAHT-MAST group (*n* = 64) who had been using GAHT for at least 6 months and had undergone mastectomy at least 3 months prior. All participants were asked to complete the Symptom Checklist-90-Revised (SCL-90-R), the Body Uneasiness Test (BUT), and the World Health Organization’s Quality of Life Questionnaire- Brief Form, Turkish Version (WHOQOL-BREF-Tr).

**Results:**

We found that individuals in the untreated group had higher psychopathological symptoms and body uneasiness scores, and lower quality of life scores compared to both GAHT and GAHT-MAST groups. There was no difference in psychopathology between the GAHT-MAST group and the GAHT group, but body uneasiness scores were lower, and quality of life scores were higher in the GAHT-MAST group.

**Conclusion:**

Our study suggests that individuals receiving GAHT improved mental health, body satisfaction, and overall quality of life. Combining mastectomy with GAHT may further enhance these benefits.

## Introduction

Individuals with gender dysphoria (GD) experience a discrepancy between their assigned sex at birth and gender identity [1], necessitating various medical interventions to align the characteristics of their desired gender with their body [2]. These interventions include social transition, psychotherapies, hormonal, and surgical interventions [2]. This process, called gender-affirming therapy (GAT), aims to gradually harmonize an individual’s bodily sex characteristics with gender identity. Hormonal treatments that create desired changes in the bodily characteristics of individuals with GD are called gender-affirming hormone therapy (GAHT), and surgical interventions are called gender-affirming surgeries (GAS). These needs often require a multifaceted approach, involving various medical specialties within the health system and players outside the healthcare system such as legal procedures.

GAHT is one of the most important therapeutic interventions aimed at reconciling bodily characteristics with gender identity and mainly includes testosterone supplementation in adults with GD who were assigned female at birth (GD AFAB) [3, 4]. It has been documented by many studies that GAHT positively affects mental health [5–12], improves quality of life [13–20] and, reduces body dissatisfaction [10, 20–22], which is one of the important components of GD [23, 24]. It can be argued that the frequent preference for testosterone supplementation leading to the development of male secondary sexual characteristics among individuals with GD AFAB [25] is associated with these positive outcomes.

Although testosterone supplementation has the potential to bring about many desired changes in the body in individuals with GD AFAB, various surgical interventions are needed to align some body parts to gender identity. Individuals with GD AFAB are often able to request GAS after testosterone supplementation, which is the first gender-confirming medical intervention. In this context, it can be stated that masculinizing chest surgery is the most preferred [26] and often the first and only surgical intervention [27, 28]. Previous studies indicate that mastectomy is a well-known, effective, safe surgical intervention [29, 30]. Existing literature has indicated that mastectomy provides several important benefits in individuals with GD AFAB [31], including improvement in psychological health [32, 33], increased quality of life [31–34], and reduced body uneasiness [26, 33, 34]. Moreover, van de Grift et al. [26] stated that the positive effects of mastectomy go beyond satisfaction with the appearance of the chest. According to their study, the development of a masculine chest post-mastectomy in individuals with GD AFAB not only enhances societal perception as male but also promotes increased social participation and facilitates positive experiences. Consequently, the favorable evaluation of one’s body and the reduction of dysphoria in social situations contribute to an improved quality of life and heightened self-esteem among these individuals [26].

In this cross-sectional study, we focused on the effects of mastectomy, which is the most preferred surgical intervention by individuals with GD AFAB, as well as the effects of GAHT. We sought answers to the following questions; (1) What kind of effects does GAHT have on psychopathology, body uneasiness, and quality of life in individuals with GD AFAB? (2) Are there any additional effects of mastectomy combined with GAHT on psychopathology, body uneasiness, and quality of life of individuals with GD AFAB? We hypothesized that GAHT would positively affect psychopathology, decrease body uneasiness, and increase the quality of life of individuals with GD AFAB, and that these effects would be further strengthened by mastectomy.

## Methods

### Participants and procedures

The sample of the present study included 297 adults with GD AFAB who consecutively applied to Istanbul University-Cerrahpaşa, Cerrahpaşa Medical Faculty for GAT between March 2018 and January 2019. The inclusion criteria were as follows: (1) being over 18 years of age; (2) diagnosis of “GD in Adolescents and Adults” according to the *Diagnostic and Statistical Manual of Mental Disorders *(*DSM-5*) [1], and confirmed after several sessions with two different psychiatrists (Ş.T. and Y.K.); (3) if GAHT is used, it has been received for at least 6 months; (4) if mastectomy has been performed, at least 3 months have passed since the operation. The exclusion criteria were as follows: (1) the presence of any neurological, metabolic, endocrinological, or disorders of sex development (DSD); (2) any GAS other than mastectomy has been performed; (3) an intellectual disability; and (4) illiteracy. It can be asserted that our study carefully addresses the confounding variables observed in many studies, where different types of GAT were simultaneously assessed, and the detailed evaluation of individuals’ GAS status [14] was not thoroughly conducted.

At the initial screening for compliance with the exclusion criteria, three people were excluded because they had a hysterectomy, two were illiterate, and two had DSD. For participants with GD AFAB who used GAHT, 21 persons who received hormones for less than 6 months were also excluded. Consequently, the study protocol was completed by 269 eligible participants with GD AFAB. Before participating in the study, all participants were provided with detailed information by two of the authors (Author1 and Author4) regarding the study’s objectives, the voluntary nature of their participation, and the assurance of confidentiality.

The participants with GD AFAB included three groups: (a) subjects with GD AFAB who were not receiving GAHT and had not undergone GAS (Untreated group; *n* = 121), (b) subjects with GD AFAB who have been only using GAHT for at least 6 months (GAHT group; *n* = 84), (c) subjects with GD AFAB who have been using GAHT for at least 6 months and have had a mastectomy at least 3 months ago (GAHT-MAST group; *n* = 64). The GAHT administration practices followed by the Cerrahpaşa Medical Faculty are largely based on the standards of care guidelines of the seventh version of *WPATH,* considering the dates of the study [2]. All individuals with GD AFAB underwent bilateral nipple sparing mastectomy with a vertical or an inverted-T scar at various medical centers. Post-mastectomy complications were assessed using the question “Have you experienced any medical issues related to mastectomy surgery?”. Two patients reported infection at the surgical site, four patients reported numbness in the arm and shoulder, and two patients reported fluid collection in the armpit. They mentioned that these complications resolved within a maximum of 3 months after treatment.

### Measures

The semi-structured sociodemographic data form*,* the Symptom Checklist-90-Revised (SCL-90-R), the Body Uneasiness Test (BUT), and the World Health Organization’s Quality of Life Questionnaire- Brief Form, Turkish Version (WHOQOL-BREF-Tr), and were completed by all participants. Sociodemographic and clinical variables such as age, level of education, employment status, relationship status, and sexual orientation were collected. Sexual orientation was assessed by asking participants the following question: “How would you describe your sexual orientation?” The response choices for this question were, “Only males are attractive to me,” “Only females are attractive to me,” “Both females and males are attractive to me,” “Neither females nor males and nor the others are attractive to me,” and “other” [10].

### Symptom checklist-90-revised (SCL-90-R)

The Symptom Checklist-90-Revised (SCL-90-R) [35] is a 90-item self-report inventory that is designed to measure ten symptoms of psychopathology (Somatization, Obsessive–Compulsive, Interpersonal Sensitivity, Depression, Anxiety, Hostility, Phobic Anxiety, Paranoid Ideation, Psychoticism, and Additional) over a 1-week interval. Responses are made on a 5-point Likert-type scale (0 = *not at all* to 4 = *extremely*). The Global Severity Index (GSI) is the mean of all of the subscale scores and indicates overall psychological distress. The validity and reliability of the Turkish version of the SCL-90-R were assessed by Dağ [36], who stated that a GSI score higher than 1.00 is considered to indicate that symptoms exist at a psychopathology level.

### Body uneasiness test (BUT)

The Body Uneasiness Test (BUT) [37] is a 71-item self-report questionnaire that is used to assess body image disturbances, with responses rated on a 6-point Likert-type scale (1 = *never* to 6 = *always*). It consists of two parts: BUT*A (consisting of 34 items) measures present body uneasiness by calculating a Global Severity Index (GSI). BUT*B (consisting of 37 items) measures the focus of attention on a specific body part or function. BUT*A investigates five factors: weight phobia, body image concerns, avoidance, compulsive self-monitoring, and depersonalization, and a GSI score over 1.2 indicates a high risk of discomfort with one’s body. BUT*B scores are combined to form two global measures: the positive symptom total (PST) and the positive symptom distress index (PSDI), with higher scores indicating greater body uneasiness. The BUT has been validated in large samples of both nonclinical and clinical (suffering from eating disorders) participants and shows good psychometric properties [37]. Cronbach’s alpha for internal consistency was 0.72 in the present study.

### World health organization quality of life questionnaire—brief form, turkish version (WHOQOL-BREF-Tr)

The World Health Organization Quality of Life Questionnaire—Brief Form (WHOQOL-BREF) is an abbreviated, 26-item version of the 100-item WHOQOL-100 quality of life measure prepared by the World Health Organization. WHOQOL-BREF consists of four domains including physical health (7 items), psychological health (6 items), social relationship (3 items), and environmental health (8 items). The last two items are about general health and overall quality of life. The Turkish adaptation study of the WHOQOL-BREF was conducted by Eser et al. [38], and the number of questions increased to 27 by adding a national question to the scale (WHOQOL-BREF-Tr). Each item is rated on a 5-point Likert-type scale and the scores are transformed to 4–20, with a higher score indicating a better quality of life. The Cronbach’s alpha value for the study was 0.85.

### Data analysis

Statistical analyses were performed using the Statistical Package for the Social Sciences (SPSS) software (version 21.0). Data were first analyzed for normality using the Kolmogorov–Smirnov test. Continuous variables are expressed as mean ± standard deviation (SD) and/or medians [interquartile range (IQR)]. Student’s t-test or analysis of variance (ANOVA) was used to compare means between groups with normal data distribution. Medians were compared using the Mann–Whitney U test and the Kruskal–Wallis test. Spearman’s rank-order test and Pearson’s correlation test were used to calculate the correlation coefficients between continuous variables. Frequencies were compared using Pearson’s and Fisher’s exact tests. In the post-hoc power analysis, the power was calculated as 91.2% with a 95% confidence level and an effect size of 0.5 for the three groups comparison. The results were evaluated at a 95% confidence interval, and *p*-values < 0.05 were considered statistically significant.

## Results

### Sample characteristics

The data from 269 participants with GD AFAB were analyzed in three groups (Untreated group, GAHT group, and GAHT-MAST group). There was no significant difference between the groups in terms of sociodemographic characteristics except for age and duration of GAHT. Among the participants included in the study, the mean age of the GAHT-MAST group (26.5 ± 5.2) was significantly higher than both the GAHT group (24.2 ± 4.8; *p* = 0.015) and the untreated group (23.7 ± 4.8, *p* < 0.001). There was no significant difference in mean age between the GAHT group and the untreated group (*p* = 1). The duration of GAHT was 11.1 ± 7.8 months in the GAHT group, and 14.2 ± 10.3 months in the GAHT-MAST group (*p* = 0.012). The mean duration after a mastectomy was 13.1 ± 12.5 months in the GAHT-MAST group. Educational status, number of siblings, marital status, employment status, income levels, social health insurance, duration of real-life experience, current lifestyle, sexual orientation, and duration of GAHT of the participants included in the study are summarized in Table 1.

**Table 1:** Comparison of the sociodemographic characteristics among all participants

Characteristics	Untreated (*n* = 121)	GAHT (*n* = 84)	GAHT-MAST (*n* = 64)	*p*
	*mean* ± *SD or n (%)*			
Age, *years*	23.7 ± 4.8	24.2 ± 4.8	26.5 ± 5.2	< 0.001*
Educational status, *years*	11.8 ± 3.2	11.8 ± 3.2	12.4 ± 3.4	0.322
Number of siblings	2.5 ± 2.8	2 ± 1.6	2.1 ± 1.6	0.773
Marital status				
Single	115 (95)	77 (91.7)	57 (89.1)	0.314
In a relationship	6 (5)	7 (8.3)	7 (10.9)	
Employment status				
Employed	70 (57.9)	54 (64.3)	36 (56.3)	0.545
Unemployed	51 (42.1)	30 (35.7)	25 (43.7)	
Income levels				
< 3000 turkish liras	41 (33.9)	22 (26.2)	18 (28.1)	0.460
> 3000 turkish liras	80 (66.1)	62 (73.8)	46 (71.9)	
Social health insurance				
Yes	117 (96.7)	80 (95.2)	59 (92.2)	0.397
No	4 (3.3)	4 (4.8)	5 (7.8)	
Duration of real-life experience, *months*	108.3 ± 94.8	107.1 ± 86.7	114.1 ± 111	0.824
Current lifestyle				
Legal gender	3 (2.5)	2 (2.4)	0	0.451
Gender you want to be	118 (97.5)	82 (97.6)	100 (100)	
Sexual orientation^†^				
Heterosexual	118 (97.5)	84 (100)	64 (100)	0.716
Homosexual	1 (0.8)	0	0	
Bisexual	1 (0.8)	0	0	
Asexual	0	0	0	
Other	1 (0.8)	0	0	
Duration of GAHT, *months*	0	11.1 ± 7.8	14.2 ± 10.3	0.012

*GAHT* gender-affirming hormone therapy; *MAST* mastectomy

Post-hoc analysis results (*adjusted p value in Bonferroni correction)*

^*^Untreated vs. GAHT, *p* = 1.0; Untreated vs. GAHT-MAST, *p* < 0.001; GAHT vs. GAHT-MAST, *p* = 0.015

^†^Heterosexuality was indicated as sexual orientation to female for individuals with female-to-male GD. Homosexuality was indicated as sexual orientation to male for individuals with female-to-male GD

### Psychopathology

The SCL-90-R subscale scores in all areas, except for Somatization, were significantly higher in the untreated group than both the GAHT group and GAHT-MAST group. There was no significant difference between the GAHT group and the GAHT-MAST group in terms of SCL-90-R subscales (*p* > 0.05 for all). The comparisons of SCL-90-R subscales between groups are detailed in Table 2.

**Table 2:** Comparison of the groups according to the SCL-90-R scale scores

SCL-90-R scale	Untreated (*n* = 121)	GAHT (*n* = 84)	GAHT-MAST (*n* = 64)	*p*
	*median (IQR 25–75)*			
Somatization	0.66 (0.33–1.25)	0.54 (0.33–0.89)	0.62 (0.25–1)	0.141
Obsessive–compulsive	1.1 (0.8–1.8)	0.7 (0.3–1.3)	0.8 (0.4–1.48)	< 0.001^*^
Interpersonal sensitivity	1.33 (0.68–2)	0.66 (0.22–1.3)	0.44 (0.14–0.85)	< 0.001^†^
Depression	1.23 (0.53–1.76)	0.46 (0.15–1)	0.38 (0.15–0.9)	< 0.001^‡^
Anxiety	0.7 (0.3–1.2)	0.4 (0.2–0.78)	0.4 (0.23–0.7)	0.001^§^
Hostility	1.16 (0.5–1.83)	0.58 (0.16–1.62)	0.5 (0.16–1.29)	< 0.001^||^
Phobic anxiety	0.42 (0.14–0.71)	0.14 (0–0.57)	0.14 (0–0.28)	< 0.001^¶^
Paranoid ideation	1.16 (0.66–1.92)	0.66 (0.33–1.16)	0.5 (0.16–0.96)	< 0.001^#^
Psychoticism	0.8 (0.5–1.2)	0.4 (0.2–0.79)	0.3 (0.1–0.69)	< 0.001^**^
Additional symptoms	1 (0.42–1.42)	0.64 (0.28–1.14)	0.57 (0.18–1.14)	0.006^††^
Global severity index	0.96 (0.65–1.46)	0.55 (0.3–0.94)	0.52 (0.3–0.83)	< 0.001^‡‡^

*SCL-90-R* Symptom Checklist-90 Revised, *GAHT* gender-affirming hormone therapy, *MAST* mastectomy

Comparison 1: Untreated vs. GAHT; Comparison 2: Untreated vs. GAHT-MAST; Comparison 3: GAHT vs. GAHT-MAST

Post-hoc analysis results (*adjusted p value in Bonferroni correction)*

^*^Comparison 1, *p* < 0.001; Comparison 2, *p* = 0.005; Comparison 3, *p* = 1.0

^†^Comparison 1, *p* < 0.001; Comparison 2, *p* < 0.001; Comparison 3, *p* = 0.286

^‡^Comparison 1, *p* < 0.001; Comparison 2, *p* < 0.001; Comparison 3, *p* = 1.0

^§^Comparison 1, *p* = 0.003; Comparison 2, *p* = 0.011; Comparison 3, *p* = 1.0

^||^Comparison 1, *p* = 0.023; Comparison 2, *p* < 0.001; Comparison 3, *p* = 0.535

^¶^Comparison 1, *p* = 0.023; Comparison 2, *p* = 0.001; Comparison 3, *p* = 0.675

^#^Comparison 1, *p* < 0.001; Comparison 2, *p* < 0.001; Comparison 3, *p* = 0.410

^**^Comparison 1, *p* < 0.001; Comparison 2, *p* < 0.001; Comparison 3, *p* = 0.305

^††^Comparison 1, *p* = 0.029; Comparison 2, *p* = 0.018; Comparison 3, *p* = 1.0

^‡‡^Comparison 1, *p* < 0.001; Comparison 2, *p* < 0.001; Comparison 3, *p* = 1.0

### Body image

The untreated group’s scores on all BUT*A subscales (Weight Phobia, Body Image Concern, Avoidance, Compulsive Self-Monitoring, Depersonalization, and Global Severity Index) were significantly higher than those of both the GAHT group and GAHT-MAST group (*p* < 0.001 for all). In the GAHT group, all BUT*A subscale scores, except for Compulsive Self-Monitoring, were significantly higher than the GAHT-MAST group.

In terms of subscales of BUT-B, BUT*B–V, and PSDI scores were significantly higher in the untreated group than in the other two groups, while they were significantly higher in the GAHT group than in the GAHT-MAST group. The BUT*B–VIII scores were significantly higher in the untreated group than the other two groups, while they were similar between the GAHT group and GAHT-MAST group. In terms of BUT*B–II, BUT*B–IV, BUT*B–VII, and PST scores, while it was determined that the untreated group had higher scores than the GAHT-MAST group and did not differ from the GAHT group, there was no significant difference between the GAHT group and GAHT-MAST group. There was no difference between the groups in terms of BUT*B–I, BUT*B–III, and BUT*B–VI (*p* > 0.05 for all). The comparisons of BUT*A and BUT*B scores between groups are shown in Table 3.

**Table 3:** Comparison of the groups according to the BUT scale scores

BUT scale	Untreated (*n* = 121)	GAHT (*n* = 84)	GAHT-MAST (*n* = 64)	*p*
	*median (IQR 25–75)*			
BUT*A				
Weight phobia	2.87 (2.37–3.63)	2.25 (1.5–3.12)	1.69 (1.12–2.72)	< 0.001^*^
Body image concern	4.11 (3.66–4.53)	3.33 (2.03–4)	1.55 (0.88–2.55)	< 0.001^†^
Avoidance	2.66 (1.75–3.33)	1.83 (0.83–2.79)	0.63 (0.16–1.96)	< 0.001^‡^
Compulsive self-monitoring	1.8 (1.2–2.8)	1.4 (0.8–2.2)	1.1 (0.6–2)	< 0.001^§^
Depersonalization	2.83 (2.32–3.25)	2 (1.16–2.83)	0.83 (0.33–1.66)	< 0.001^||^
Global severity index	3 (2.5–3.6)	2.34 (1.48–2.96)	1.32 (0.75–2.07)	< 0.001^¶^
BUT*B				
BUT*B–I (eyebrows, eyes, nose, mouth, lips, teeth)	0.5 (0–0.83)	0.16 (0–0.79)	0.16 (0–0.5)	0.076
BUT*B–II (shape of the head and face, forehead, ears, chin, neck)	0 (0–0.66)	0 (0–0.79)	0 (0–0.33)	< 0.029^#^
BUT*B–III (stomach, abdomen, hips, thighs, knees)	1.2 (0.4–2.2)	0.8 (0.05–1.8)	0.9 (0.25–1.8)	0.083
BUT*B–IV (stature, legs, ankles, feet, hands)	0.6 (0.1–1.8)	0.4 (0–1.2)	0.3 (0–0.95)	0.013^**^
BUT*B–V (arms, shoulders, chest, breasts, genitals)	3 (3–3.6)	3 (2.65–3)	1.4 (1–2)	< 0.001^††^
BUT*B–VI (mustache, beard, facial hair)	0 (0–1)	0 (0–0.25)	0 (0–0.92)	0.332
BUT*B–VII (hair, skin)	0.5 (0–2)	0.25 (0–1)	0 (0–0.5)	0.009^‡‡^
BUT*B–VIII (sweating, blushing, noises, odors, buttocks)	1.8 (1–2.2)	1 (0.4–1.6)	1 (0.6–1.6)	< 0.001^§§^
PST	13 (8–17.5)	10 (7–17)	9 (5–15)	0.013^||||^
PSDI	3.6 (3.11–4.16)	3.16 (2.56–3.91)	2.5 (2–3.6)	< 0.001^¶¶^

*BUT* Body Uneasiness Test, *GAHT* gender-affirming hormone therapy, *MAST* mastectomy

Comparison 1: Untreated vs. GAHT; Comparison 2: Untreated vs. GAHT-MAST; Comparison 3: GAHT vs. GAHT-MAST

Post-hoc analysis results (*adjusted p value in Bonferroni correction)*

^*^Comparison 1, *p* = 0.001; Comparison 2, *p* < 0.001; Comparison 3, *p* = 0.036

^†^Comparison 1, *p* < 0.001; Comparison 2, *p* < 0.001; Comparison 3, *p* < 0.001

^‡^Comparison 1, *p* = 0.001; Comparison 2, *p* < 0.001; Comparison 3, *p* = 0.001

^§^Comparison 1, *p* = 0.010; Comparison 2, *p* < 0.001; Comparison 3, *p* = 0.187

^||^Comparison 1, *p* < 0.001; Comparison 2, *p* < 0.001; Comparison 3, *p* < 0.001

^¶^Comparison 1, *p* < 0.001; Comparison 2, *p* < 0.001; Comparison 3, *p* < 0.001

^#^Comparison 1, *p* = 1.0; Comparison 2, *p* = 0.036; Comparison 3, *p* = 0.076

^**^Comparison 1, *p* = 0.092; Comparison 2, *p* = 0.022; Comparison 3, *p* = 1.0

^††^Comparison 1, *p* = 0.006; Comparison 2, *p* < 0.001; Comparison 3, *p* < 0.001

^‡‡^Comparison 1, *p* = 1.0; Comparison 2, *p* = 0.007; Comparison 3, *p* = 0.131

^**§§**^Comparison 1, *p* < 0.001; Comparison 2, *p* < 0.001; Comparison 3, *p* = 1.0

^**||||**^Comparison 1, *p* = 0.297; Comparison 2, *p* = 0.011; Comparison 3, *p* = 0.595

^¶¶^Comparison 1, *p* = 0.010; Comparison 2, *p* < 0.001; Comparison 3, *p* = 0.005

### Quality of life

About the scores of the WHOQOL-BREF-Tr subscales, there was no significant difference between the groups in terms of physical health (*p* = 0.32), but there was a significant difference in terms of psychological (*p* < 0.001), social relationships (*p* < 0.001), environment (*p* = 0.002), and general health (*p* < 0.001). While the untreated group had significantly lower scores on only psychological subscales than the GAHT group, they had significantly lower scores on all subscales except physical health compared to the GAHT-MAST group. In addition, psychological and general health subscale scores were found to be significantly lower in the GAHT group compared to the GAHT-MAST group (respectively, *p* < 0.001; *p* = 0.002). (Table 4).

**Table 4:** Comparison of the groups according to the WHOQOL-BREF-Tr scale scores

WHOQOL-BREF-Tr scale	Untreated (*n* = 121)	GAHT (*n* = 84)	GAHT-MAST (*n* = 64)	*p*
	*median (IQR 25–75)*			
Physical health	23 (21–25)	23 (22–26)	24 (21–26)	0.323
Psychological	17 (15–19.5)	19 (16.25–21)	22 (20–24)	< 0.001^*^
Social relationships	11 (8.5–12)	12 (10–13)	12 (11–14)	< 0.001^†^
Environment	28 (25–31)	29 (25–31.75)	31 (27–35)	0.002^‡^
General health	7 (6–7)	7 (6–8)	8 (7–8.75)	< 0.001^§^

*WHOQOL-BREF-Tr* World Health Organization Quality of Life Assessment – Brief Form, Turkish Version, *GAHT* gender-affirming hormone therapy, *MAST* mastectomy

Comparison 1: Untreated vs. GAHT; Comparison 2: Untreated vs. GAHT-MAST; Comparison 3: GAHT vs. GAHT-MAST

Post-hoc analysis results (*adjusted p value in Bonferroni correction)*

^*^Comparison 1, *p* = 0.014; Comparison 2, *p* < 0.001; Comparison 3, *p* < 0.001

^†^Comparison 1, *p* = 0.058; Comparison 2, *p* < 0.001; Comparison 3, *p* = 0.152

^‡^Comparison 1, *p* = 0.640; Comparison 2, *p* = 0.001; Comparison 3, *p* = 0.081

^§^Comparison 1, *p* = 0.762; Comparison 2, *p* < 0.001; Comparison 3, *p* = 0.002

### Correlations

When the correlations between the duration of real-life experience and the scores of all scales in participants were examined, there was only a weak negative correlation with the SCL-90-R somatization subscale (*r* =  − 0.155, *p* = 0.011). There was no significant correlation between the duration of hormone use and the scores of all the subscales of the participants using hormones (*p* > 0.05 for all). There was no correlation between the time after mastectomy and the scale scores of the participants who had a mastectomy (*p* > 0.05).

## Discussion

In this study, we investigated alterations in psychopathology, body uneasiness, and quality of life after the use of GAHT and mastectomy added to GAHT in a sample of participants with GD AFAB. The main results were as follows: (1) psychopathological symptoms of people with GD AFAB were significantly lower in both GAHT and mastectomy added to GAHT compared to individuals who did not receive any GAS, but there was no additional positive effect of mastectomy; (2) while body uneasiness scores were lower in individuals with GD AFAB who received GAHT than in those who did not, in individuals who had a mastectomy added to GAHT, this uneasiness was further reduced in individuals who had mastectomy added to GAHT compared to those who received only GAHT; (3) in terms of quality of life, individuals with GD AFAB who received GAHT had higher scores on only psychological subscales than those who did not, while those who had additional mastectomy to GAHT had higher scores on all subscales except physical health compared to those who did not receive GAHT.

Individuals with GD face many negative situations throughout their lives that make them more vulnerable to mental health problems [39, 40]. This vulnerability is rooted in several contributing factors, including the distress induced by the incongruence between physical/biological characteristics and gender identity, the prolonged and challenging process of GAT, and the phenomenon of “minority stress,” which has the potential to manifest in diverse physical and psychological adversities [41, 42]. Not surprisingly, given all these vulnerability conditions, we also found that all psychopathology scores except Somatization were higher in the untreated group. A recent systematic review [43] indicates that individuals with GD suffer severely from psychiatric disorders, particularly mood disorders (42.1%), anxiety disorders (26.8%), and substance-related disorders (14.7%). On the other hand, numerous studies [7, 8, 10–12, 44, 45] highlight that GAHT is an important treatment option in solving the distress caused by the incongruency of physical/biological characteristics and gender identity. In our study, according to the literature, lower levels of psychopathology were found in the GAHT group. Also, few studies [31, 33, 46] conducted with individuals with GD AFAB indicate that mastectomy has positive effects on the mental health of these individuals. However, it can be stated that the effects of GAHT have not been adequately examined in these studies. Although it was stated in the prospective study of Agarwal et al. [31] that 93% of individuals (n = 42) who underwent mastectomy were using hormones, there is no information about the duration of GAHT. In another prospective study by Lane et al. [33], no information about GAHT was found. In addition, Van de Grift et al. reported that mastectomy in individuals with GD AFAB was associated with higher levels of postoperative psychological function satisfaction [47]. The findings of our study indicate that mastectomy does not have significant positive effects on mental health in addition to GAHT. Considering that there are many factors affecting mental health in individuals with GD, these results should be interpreted carefully. In this context, it can be concluded that the effects of mastectomy on the mental health of individuals with GD AFAB in addition to GAHT should be investigated with larger samples and longitudinal studies.

Some researchers have emphasized that the primary source of distress in GD is concerns about the body [48, 49]. In this context, it can be said that eliminating concerns about the body in GD is an important goal of medical interventions. Previous studies report that GAHT or GAS may help address people with GD’s uneasiness with their bodies [5, 10, 20, 45, 50, 51]. The findings of our study, consistent with this evidence from research, indicate that GAHT reduces all features that reflect body uneasiness and are included in BUT*A (weight phobia, body image concern, avoidance, compulsive self-monitoring, depersonalization) in individuals with GD AFAB. In addition, in terms of BUT*B, which evaluates body regions, we found that GAHT reduced uneasiness in BUT*B–V (arms, shoulders, chest, breasts, genitals) and BUT*B–VIII (sweating, blushing, noises, odors, buttocks). Considering that these body regions are connected to where dysphoria is somatically experienced the most [52] and where the greatest change is observed with the use of GAHT, it can be stated that this result is not a surprise.

In our study, we found that all BUT*A subscale scores except compulsive self-monitoring were significantly lower in individuals with GD AFAB who underwent mastectomy in addition to GAHT, compared to those who used GAHT alone. In the prospective study of Agarwal et al. [31], who used BUT-A, it was shown that the preoperative scores of all subscales decreased significantly in the 6th month of the postoperative period. In the prospective follow-up study conducted by van de Grift et al. [26], it is emphasized that the effects of mastectomy on body image are not limited to satisfaction with the chest area and have a broader impact. Another finding of our study is that BUT*B–II (shape of the head and face, forehead, ears, chin, neck), BUT*B–IV (stature, legs, ankles, feet, hands), BUT*B–V (arms, shoulders, chest, breasts, genitals) and BUT*B–VII (hair, skin) scores decreased even more after mastectomy in addition to the effects of GAHT, indicates this broad impact area. It can be said that the decrease in weight phobia, body image concern, avoidance, and depersonalization after mastectomy, as well as the decrease in negative perceptions about many body parts, is associated with a more positive evaluation of the body, a decrease in dysphoria in social situations and an increase in the quality of life [26].

Previous meta-analysis studies indicate that the quality of life of individuals with GD is lower than the general population [53] and that GAHT improves the quality of life of these individuals [13, 14]. However, it was emphasized that the findings should be interpreted with caution due to the high risk of bias in the study designs, small sample sizes, and other confounding interventions [14]. Our findings show that only the psychological subscale scores of individuals using GAHT are higher in terms of quality of life compared to those who do not use GAHT, and also that the social relationships subscale scores approach significance (*p* = 0.058). These results are consistent with the results of meta-analyses stating that GAHT has an improving effect on the quality of life of individuals with GD. In our study, it was observed that the GAHT group used hormones for an average of 11.1 months. Considering that the physical effects of GAHT continue to occur within 2–5 years [2], it can be stated that the possibility that the positive effects of GAHT may increase over time should be taken into consideration. One of the important findings of this study is that mastectomy has been shown to have positive effects on all quality of life subscales except one (physical health), compared to untreated individuals. Previous studies have shown that mastectomy has quality of life improving effects in individuals with GD AFAB [31–34]. However, since it was not clear how long the participants had been using GAHT in those studies, it can be thought that the effect of mastectomy could not be fully evaluated. The present study shows that among individuals with GD AFAB who received GAHT for a similar duration, those who underwent mastectomy had a better quality of life. This suggests that mastectomy further extends the positive effects of GAHT in terms of quality of life in individuals with GD AFAB.

There were several limitations to this study. Firstly, its cross-sectional nature requires careful consideration of our interpretations regarding causal relationships. Additionally, the absence of a cisgender control group made it impossible to compare the results with the general population. Furthermore, there was a lack of detailed information about post-mastectomy processes. Lastly, self-report measures were used, which may have been subject to social desirability bias.

## Conclusion

To sum up, GAHT and mastectomy have been shown to have a positive impact on the mental health of individuals with GD AFAB, reducing body uneasiness and improving their quality of life. The results of the study suggest that those who received GAHT experienced fewer mental health issues, were more content with their bodies, and had a better quality of life compared to those who did not receive GAHT. Furthermore, combining mastectomy with GAHT could enhance these positive effects even further. To gain a better understanding of the impact of medical interventions on mental health, body satisfaction, and quality of life in individuals with GD, future studies should have larger samples and long-term follow-up.

## Data Availability

The datasets generated and/or analyzed during the present study are available from the corresponding author on reasonable demand.
